# Can the Cognitive Phenotype in Neurofibromatosis Type 1 (NF1) Be Explained by Neuroimaging? A Review

**DOI:** 10.3389/fneur.2019.01373

**Published:** 2020-01-14

**Authors:** Eloïse Baudou, Federico Nemmi, Maëlle Biotteau, Stéphanie Maziero, Patrice Peran, Yves Chaix

**Affiliations:** ^1^Children's Hospital, Purpan University Hospital, Toulouse, France; ^2^ToNIC, Toulouse NeuroImaging Center, University of Toulouse, Inserm, UPS, Toulouse, France; ^3^Octogone-Lordat, University of Toulouse, Toulouse, France

**Keywords:** NF1, cognitive phenotype, cerebral substrate, brain imagery, fMRI

## Abstract

Neurofibromatosis type 1 (NF1) is one of the most frequent monogenetic disorders. It can be associated with cognitive dysfunctions in several domains such as executive functioning, language, visual perception, motor skills, social skills, memory and/or attention. Neuroimaging is becoming more and more important for a clearer understanding of the neural basis of these deficits. In recent years, several studies have used different imaging techniques to examine structural, morphological and functional alterations in NF1 disease. They have shown that NF1 patients have specific brain characteristics such as Unidentified Bright Objects (UBOs), macrocephaly, a higher volume of subcortical structures, microstructure integrity alterations, or connectivity alterations. In this review, which focuses on the studies published after the last 2 reviews of this topic (in 2010 and 2011), we report on recent structural, morphological and functional neuroimaging studies in NF1 subjects, with special focus on those that examine the neural basis of the NF1 cognitive phenotype. Although UBOs are one of the most obvious and visible elements in brain imaging, correlation studies have failed to establish a robust and reproducible link between major cognitive deficits in NF1 and their presence, number or localization. In the same vein, the results among structural studies are not consistent. Functional magnetic resonance imaging (fMRI) studies appear to be more sensitive, especially for understanding the executive function deficit that seems to be associated with a dysfunction in the right inferior frontal areas and the middle frontal areas. Similarly, fMRI studies have found that visuospatial deficits could be associated with a dysfunction in the visual cortex and especially in the magnocellular pathway involved in the processing of low spatial frequency and high temporal frequency. Connectivity studies have shown a reduction in anterior-posterior “long-range” connectivity and a deficit in deactivation in default mode network (DMN) during cognitive tasks. In conclusion, despite the contribution of new imaging techniques and despite relative advancement, the cognitive phenotype of NF1 patients is not totally understood.

## Introduction

In recent years, the cognitive and behavioral phenotypes of Neurofibromatosis 1 (NF1) have been well described in affected children. Despite some heterogeneity, which is not yet clearly understood in the absence of established genotype-phenotype correlation, the main cognitive characteristics that have been highlighted are:

- On average, an intelligence quotient (IQ) score lower by 1 standard deviation (SD) compared to the general population (1) with intellectual deficit for about 6% of the NF1 patients.
- Visuospatial impairment, highlighted in particular with the Benton Judgment of Line orientation test (JLO) (1).
- Language disabilities in about 50% of the cases, especially in phonological processes (2).
- Attention deficit according to the diagnostic criteria for Attention Deficit and Hyperactivity Disorder (ADHD) in 30 to 40% of the cases (3, 4), and in general, executive function deficits (5).
- Social cognition deficit, sometimes with the Autism Spectrum Disorder (ASD) criteria (6).
- Motor coordination disorder (7).

Therefore, different cognitive domains are affected, which suggests that different brain networks are involved in the physiopathology of NF1.

However, NF1 patients present specific neuroimaging features. Among them, Unidentified Bright Objects (UBOs) are the best known and are suggested as diagnostic criteria by some authors (8). However, the presence of UBOs does not explain the cognitive and behavioral phenotype in NF1 disease (9, 10). A recent study using multimodal neuroimaging including structural, diffusion and resting state functional magnetic resonance imaging (fMRI) (11) showed that NF1 patients and healthy controls can be differentiated using neuroimaging that combines the measurement of gray matter volume, fractional anisotropy and mean diffusivity. This suggests a complex physiopathology involving gray and white matter abnormalities.

The links between behavioral and cognitive phenotypes and the cerebral substratum are poorly understood. However, the number of brain imaging studies has considerably increased since the reviews of literature by Payne et al. (9) and Hachon et al. (10).

Based on the evidences established in the two previous reviews, we pursued an examination of the literature since 2010 concerning brain imaging in NF1 disease. The aim of this literature review is to find out whether in 2019, nearly 10 years after the last reviews, there is a clearer understanding of the link between cognitive and behavioral patterns and NF1 cerebral physiopathology in children with NF1.

## Review

### Structural Level

#### Summary of the Findings Before 2010

On average, NF1 patients have a larger brain volume than the general population, with macrocephalia in 50% of the cases. This difference in brain volume is predominant in white matter (WM), and less significant in gray matter (GM) (12). Up until now, results from brain imaging studies have been unable to clearly indicate any correlation between total brain volume, WM volume or GM volume and the neuropsychological profile. For example, the majority of studies report an NF1-related increase in corpus callosum (CC) volume (13, 14), sometimes with a positive correlation with learning disability (13) and sometimes a negative correlation with ADHD (14) (Table 1).

**Table 1:** Correlation between Structural MRI analysis and Neuropsychological findings: main characteristics of the neuroimaging studies included.

**References**	**Participants**	**Mean age (SD); range**	**Neuroimaging acquisition**	**Neuroimaging results**	**Neuropsychological correlations**
Moore et al. (13)	52 NF1 19 controls	9,8 (3,6); r: 3-16,9 10,9 (2,7); r: 7-16	MRI T1	Higher volume of GM	Learning disability
				Larger CC size	Academic achievement and visual-spatial and motor-skills
Kayl et al. (14)	36 NF1 18 controls	10,6 (2,8); r: 6-16 11,0 (2,7); r: 6-16	MRI T1	Larger total CC area	Less attention problems
Billingsley et al. (15)	24 NF1 24 controls	10,8; r: 7,4-15,8 11,5; r: 7-16,6	MRI T1	Less leftward asymmetry	Poorer reading and math achievement
Billinsley et al. (16)	38 NF1 38 controls	10,8; r: 7,4-15,8 11,5; r: 7-16,6	MRI T1	Right IFG: typical pattern	Worse for all language measurements (phonologic fluency, verbal knowledge, reading, spelling and verbal memory)
				Heschl's gyrus: doubling in left hemisphere	Poorer performance on verbal memory, fine motor speed and coordination measurements
				Heschl's gyrus: doubling in right hemisphere	Better performance on math, verbal memory and fine motor speed
Huijbregts et al. (17)	15 NF1 18 controls	12,9 (2,6) 13,8 (3,6)	MRI T1	Larger left putamen volume and larger total WM volume	More social problems and poorer executive functioning
				Larger right amygdala volume	Poorer executive functioning and autistic mannerisms
Aydin et al. (18)	37 NF1 31 controls	12,9 (2,6) 9,83 (3,76)	MRI DWI: ADC	Genu CC: higher ADC values	Poorer arithmetic, digit span and coding scores
			MRI DWI: FA	Genu CC: higher FA values	Poorer coding scores
Koini et al. (19)	16 NF1 32 controls	12,45 (2,75); r: 9,3-18,6 12,43 (2,99); r: 9,2-19,0	MRI DWI: FA MD AD RD	Altered whole brain microstructure (MD and AD) and ATR; but not CB and SLF	Poorer executive functioning: inhibitory control. No correlation with verbal and performance abilities

*AD, axial diffusion; ADC, apparent diffusion coefficient; ATR, anterior thalamic radiation; CB, cingulate bundle; CC, corpus callosum; DWI, diffusion weighted images; FA, fractional anisotropy; GM, gray matter; IFG, inferior frontal gyrus; MD, mean diffusivity; MRI, magnetic resonance imaging; NF1, neurofibromatosis type 1; r, range; RD, radial diffusion; SD, standard deviation; SLF, superior longitudinal fasciculus; WM, white matter*.

Billingsley et al. highlighted focal architectural abnormalities in NF1 (15, 16). They showed a link between language disability/low academic achievement (reading and/or mathematics) and reduced asymmetry of the left/right planum temporale (Table 1). They also found a positive interaction between an atypically structured right inferior frontal gyrus and language level (Table 1). These studies suggest an atypical lateralization of linguistic functions in NF1 subjects.

#### Post-2010 Studies: From the Macrostructural to the Microstructural Level

In an NF1 group, Huijbregts et al. (17) found a larger WM volume associated with a larger volume of all subcortical structures (hippocampus, thalami, striatum, amygdala, accumbens nucleus) and a lower GM density in frontal and parietal regions (Table 1). Positive correlations were found between cognitive abilities and social skills, and the volume of subcortical structures:

- Right amygdala volume correlated with executive functions assessed with the Behavioral Rating Inventory of Executive Function (BRIEF) and autistic behavior assessed with the Social Responsiveness Scale (SRS).
- Left putamen volume correlated with executive functions assessed with the Dysexecutive Questionnaire (DEX) and social problems assessed with the Child Behavior Check List (CBCL).

Violante et al. (20) also found that subcortical structures (thalami, right caudate, middle CC) had larger volumes in a group of 14 NF1 children aged 8 to 16 years compared to 14 controls. In this study, the authors confirmed a larger whole brain volume (+10%) with a greater difference between WM (+20%) and GM (+8%) compared to controls. They analyzed the volume variation in more detail according to the function of the region and found a greater difference in bilateral frontal and temporal regions and in the left parietal region for lobar WM volumes. They observed less distinctive gyrification in NF1 subjects, without any difference in cortical volume, cortical surface or cortical thickness. In human phylogenic evolution, an increase in brain volume is associated with an increase in cortical gyrification (21). However, in NF1 patients, gyrification is not proportional to brain volume.

Aydin et al. (18) found a higher CC volume in NF1 children (Table 1). This study focused on micro- and macrostructural measurements of the CC (midsagittal CC area measurements, fractional anisotropy (FA), and absolute diffusion coefficient (ADC) values of the genu and splenium of the CC). Negative correlations were shown between the ADC values of the genu of the CC and the arithmetic and digit span scores and between the FA values in the genu and coding scores in children with NF-1.

Karlsgodt et al. (22) compared 14 young adults with NF1 and 12 healthy controls using Diffusion Tensor Imaging (DTI) analyses. These authors confirmed an increase in WM volume in NF1 patients and showed an alteration in WM integrity in the anterior thalamic radiation (ATR). This study specifically showed a decrease in FA and an increase in ADC and radial diffusivity (RD) and, to a lesser extent, in axial diffusivity (AD) in the ATR. This pattern suggests an increase in diffusivity due to reduced myelination and reduced axonal organization. More recently, Koini et al. (19) showed a correlation between a decrease in executive functions (inhibitory control evaluated by a sustained attention test) and modifications in microstructure parameters (a decrease in FA and an increase in mean diffusivity, RD and AD) in the ATR (Table 1). It is noteworthy that there was no link between these alterations and the presence of thalamic UBOs.

##### Summary

In comparison to the general population, NF1 subjects present an increase in brain volume that is more pronounced in WM than in GM. However, cortical gyrification is proportionally less compared to healthy subjects. Similarly, the volume of the CC, the thalamus and the striatum is larger in the NF1 population. At the microstructural level, a decrease in FA and an increase in mean diffusivity seem to be systematic, possibly due to an alteration in myelination. Finally, a link can be established between abnormalities in the ATR and the executive dysfunction observed in NF1 subjects. Characteristics of the main neuroimaging studies that show a link between structural features and cognitive functions are indicated in Table 1.

### UBOs

#### Summary of Findings Before 2010

UBOs are an anatomical feature of the brain of NF1 children and adults. However, they are not considered to be a criterion in the diagnosis of NF1. UBOs are hyperintensities on T2-weighted or Fluid-Attenuated Inversion Recovery (FLAIR) MRI sequences, without mass effect, and without contrast enhancement. We will interchangeably use the term UBOs and T2-hyperintensities in the following paragraphs. UBOs are found in approximately 70% of NF1 subjects (23), but only between 0.8 and 2.2% in the general population, depending on the study. UBOs can be discrete or diffuse. They are principally located in basal ganglia, thalami, cerebellum and brainstem. In less than 20% of the cases UBOs are supratentorial and hemispheric. They tend to regress with age, at least for those located in the basal ganglia and the brainstem (24). There are different hypotheses concerning their nature: e.g., low grade tumor, hamartoma, heterotopias or a modification in the water content of myelin with dysplastic glial cells. Some authors used MRI to try to clarify the microstructural nature of UBOs. Using DTI, they showed an increase in ADC (25, 26) and a decrease in FA (27). The only anatomo-pathological study of UBOs was conducted by Dipaolo et al. (28). Their histological analysis showed that UBOs result from a vacuolar and spongiotic alteration in WM caused by intramyelinic edema. However, a limitation of their study is the heterogeneity of the NF1 population investigated, since one subject was born prematurely and another received chemotherapy for fibrosarcoma.

The most important question concerning UBOs is their possible involvement in cognitive impairment and their impact on learning, especially in NF1 children. The literature does not provide a definitive answer, even though some studies have highlighted the importance of the location of UBOs in cognition rather than their numbers, with a possible link between thalamic location and cognitive impairment (IQ, attention span) (29). Feldman et al. (30) showed a link between a decrease in T2-hyperintensities in basal ganglia (thalami) and IQ point gain (Table 2).

**Table 2:** Correlation between the MRI analysis of UBOs and neuropsychological findings: main characteristics of the neuroimaging studies included.

**References**	**Participants**	**Mean age (SD); range**	**Neuroimaging acquisition**	**Neuroimaging results**	**Neuropsychological correlations**
Moore et al. (29)	84 NF1	12,04; r: 8-16	MRI T2	Thalamic T2H location	IQ, memory, motor, distractibility and attention
Feldman et al. (30)	67 NF1 20 controls	16,3 (8,7); r: 6-3716,6 (8,6); r: 6-39	MRI T2	Decreased T2H (basal ganglia, thalamus) on 3-year follow up	Improved IQ (+8 points)
Payne et al. (31)	18 NF1 5 controls	12,4 (2,5); r: 8-16,812,0 (2,3); r: 8,9-15,2	MRI T2	Decreased T2H on 18-year follow up	Improved IQ
Piscitelli et al. (32)	49 NF1	10,2 (2,9); r: 6-16,9	MRI T2	Cerebellar T2H location	Lower scores for subtest information and vocabulary on the WISC-III, arithmetic and vocabulary, total IQ, fluid reasoning IQ
Roy et al. (33)	36 NF1	9,62 (1,74); r: 7-12,92	MRI T2	Number, size, location T2H	No correlation with executive functions and IQ

*IQ, intellectual quotient; MRI, magnetic resonance imaging; NF1, neurofibromatosis type 1; r, range; SD, standard deviation; T2H, T2 hyperintensity; UBOs, unidentified bright objects; WISC-III, Wechsler Intelligence Scale for Children III*.

#### Post 2010 Studies

##### Morphologic neuroimaging and the relationship with cognitive phenotype

In a large cohort, Sabol et al. (8) confirmed the presence of UBOs in 73.5% of 162 NF1 children aged 2 to 18 years, vs. 4.3% of 163 healthy controls. This provides excellent specificity for the diagnosis of NF1 when UBOs are present (specificity: 98%, sensitivity: 81% before 7 years of age). This study confirmed that the basal ganglia were the most frequent location of T2-hyperintensities and that they decrease with age. Payne et al. (31) highlighted this decrease in T2-hyperintensities through a longitudinal study in which the authors presented cognitive (IQ) and structural neuroimaging data (UBOs) (Table 2). They showed a decrease of 35% in T2-hyperintensities over an 18-year period, with differences in progression depending on the type of lesion (discrete lesions decreased and diffuse lesions remained unaltered) and the location (deep lesions in the basal ganglia, cerebellum, and brainstem decreased while hemispheric lesions remained unaltered). A decrease in UBOs was associated with an increase in IQ only on the third assessment, while IQ remained stable in subjects without T2-hyperintensities.

Piscitelli et al. (32) showed a relationship between cerebellum T2-hyperintensities and the neurocognitive profile. Subjects with cerebellar UBOs (31 out of 49 NF1 children in the study) presented worse scores on verbal IQ, full-scale IQ and visuospatial tests (reasoning and memory) than subjects without cerebellar hyperintensities (18 out of 49 NF1 children in the study). However, Roy et al. (33) showed no relationship between executive functions, evaluated with a test or a questionnaire, and the presence, number or location of T2-hyperintensities (Table 2).

##### Diffusion imaging for a better understanding of UBOs

MRI, especially DTI sequences, has been used in several studies to further the understanding of the microstructure of UBOs. Ferraz-Filho et al. (34) showed a decrease in FA values in the bilateral cerebellum and thalami in NF1 patients, regardless of the occurrence of UBOs in the thalami. This suggests that microstructural abnormalities can be present even if there are no hyperintensities in the brain. In a second study (35) with 27 NF1 subjects on whom 2 MRI examinations were performed between 1 and 5 years, the authors confirmed a decrease in T2-hyperintensities with a non-linear pattern of progression after the first decade of life. During the first decade of life, hyperintensities can remain stable or increase in number. The author found a reduction in the mean FA in UBOs regions and in regions where UBOs have disappeared (i.e., thalami, cerebellum and basal ganglia).

To better understand the microstructural modifications in UBOs sites, Billiet et al. (36) combined DTI analysis with other MRI-based techniques such as multi-exponential T2 relaxation, diffusion kurtosis imaging or neurite orientation dispersion and density imaging. They compared these parameters in 17 NF1 subjects, within UBOs sites and in contralateral normal-appearing WM. The authors found a lower FA, greater mean diffusivity (MD), RD and AD, and a longer T2 time for intracellular and extracellular water in UBOs in comparison to contralateral normal appearing WM. The authors considered that these results might have been related to intramyelinic edema. Ertan et al. (37) analyzed DTI parameters (FA, MD, AD and RD) in regions of interest in 14 NF1 subjects and 14 healthy controls, comparing UBOs sites and normal appearing sites. The decrease in FA was found in GM and WM UBOs, but mostly in WM. Previous studies suggested that a combined decrease in FA and increase in AD and RD could be explained by a combination of myelin damage and axonal disturbance. Tractography showed WM fiber integrity in 15 UBOs out of 18.

##### Multimodal approach

Barbier et al. (38) compared spectroscopic imaging in a multivoxel approach in basal ganglia and thalami in 25 NF1 children aged 8 to 15 years divided into two groups, one without UBOs (UBOs − group: 10 subjects) and one with UBOs (UBOs + group: 15 subjects). These authors found lower N-acetyl-aspartate (NAA)/creatinine, NAA/Choline and NAA/myoInositol ratios and a higher MyoInositol/Choline ratio in the right lateral thalamus in the UBOs + group, compared with the UBOs − group. These results could suggest a thalamic dysfunction that affects the thalamo-cortico-frontal loops related to neural and/or astroglial abnormalities. In a multimodal approach that combines spectroscopy MRI and DTI, Nicita et al. (39) analyzed spectroscopy imaging and 2 DTI parameters (ADC and FA) for 4 regions of interest (the caudate nucleus, the globus pallidus, the putamen and the thalamus) in 14 NF1 subjects aged 8 to 31 years and 8 healthy controls. The authors found (1) lower NAA, NAA/choline and NAA/creatinine ratios regardless of the subject's age (under or above 18 years of age) and the presence or absence of UBOs when the NF1 subjects were compared with the controls; (2) and a higher ADC without FA changes in UBOs subjects and subjects under 18 years of age. The presence of metabolic and microstructural abnormalities was an indication of axonal damages associated with an increase in myelin turnover in areas of intramyelinic edema, especially in young subjects. Interestingly, the subjects in this study manifested no developmental delay or cognitive deficits. These results somewhat contradict those of Rodrigues et al. (40), who found a preservation of NAA values but an increase in MyoInositol/Creatinine and Choline/Creatinine ratios in the basal ganglia with the use of a larger sample (42 NF1 subjects aged 4 to 24 years and 25 healthy controls) regardless of the UBOs status (presence or absence). Lastly, Violante et al. (41) used magnetic resonance spectroscopy (MRS) and [11C]-flumazenil PET, to compare 14 NF1 adults and 13 matched controls. These authors found a lower gamma-aminobutyric acid (GABA) concentration in the visual cortex and the frontal eye fields (FEF) (11.5 and 22% respectively), and a reduction in the binding of GABA_A_ receptors in the left parieto-occipital cortex, midbrain and thalami, which were not explained by a lower GM volume. Only a correlation between GABA concentration and GABA_A_ receptor density was found.

#### Summary

UBOs are present in almost ¾ of the NF1 children, with the basal ganglia being the most frequent anatomical location. UBOs decrease in number after the first decade and this decrease is associated by cognitive improvement. This decrease affects discrete lesions (vs. diffuse) and deep lesions (vs. hemispheric). The microstructural studies found lower FA and higher MD, RD, AD, and mean T2 time, which supports the notion of a myelin edema. These microstructural abnormalities persist after UBOs regression, which indicates that structural abnormalities exist with or without macroscopic lesions (UBOs). Studies using MRS confirm these results but are contradictory with regards to the axonal damage associated with myelin damage. Characteristics of main neuroimaging studies showing a link between UBOs and cognitive functions are indicated in the Table 2.

### Functional Level

#### Summary of the Findings Before 2010

Few studies used functional brain imaging to investigate NF1 physiopathology before 2010. Studies with Positron Emission Tomography (PET) scans that utilized [18F] fluorodeoxyglucose (FDG) usually included a small number of subjects (<30). They suggested thalamic hypometabolism in 9 NF1 children [Kaplan et al. (42)] and 29 NF1 adults [Buchert et al. (43)] compared to matching healthy controls.

Four studies published before 2010 used functional MRI (fMRI). In 2003, Billingsley's team (16) compared brain activation during a phonologic and an orthographic task in NF1 children and healthy controls (Table 3). They showed greater activation in the right hemisphere during the phonologic task in the NF1 children. They also reported greater involvement of posterior regions (middle temporal and occipital regions) than frontal regions during the orthographic tasks in the same group. The authors interpreted these results as compatible with a “disconnection” between anterior and posterior brain regions in the NF1 population, related to the WM damage that exists in this disease. In 2004, Billingsley et al. (44), the same 2 groups were compared during a letter and number identification task presented under 3 different conditions: baseline condition, mirror condition (targets were inverted) and rotation condition (targets were rotated at varying degrees). The authors found higher brain activity in posterior regions (middle temporal, parietal and occipital cortices) than in anterior regions (frontal cortices) in the NF1 subjects compared to the controls during visual-spatial analysis. They suggested that the functional abnormalities observed in this study could be related to structural abnormalities that were previously reported by the same team in these regions. Lastly, Clements-Stephens et al. (45) (Table 3) showed an inefficient right hemisphere network and more significant involvement of the left hemisphere in an NF1 group during a JLO task. The authors also found decreased activation in the primary visual cortex of the NF1 sample in comparison to the healthy controls. Shilyansky et al. (46) (Table 3) found lower activation in NF1 subjects within several cortical and subcortical regions of the right hemisphere dorsolateral prefrontal cortex, FEF and striatum during a visuospatial working memory task.

**Table 3:** Correlation between fMRI analysis and neuropsychological findings, main characteristics of the neuroimaging studies included.

**References**	**Participants**	**Mean age (SD); range**	**Neuroimaging acquisition**	**Neuroimaging results in NF1 group**	**Neuropsychological correlations in NF1 group**
Billingsley et al. (16)	15 NF1 15 controls	14,4 (4,0) 15,3 (3,7)	fMRI: visual orthographic task	Higher activation in posterior regions than in frontal regions (left inferior frontal, left DLPFC, premotor cortices)	Poorer performance in visual orthographic task
			fMRI: auditory rhyme task	Higher activation in right hemisphere (right STG)	Poorer performance in auditory rhyme task
Billingsley et al. (44)	15 NF1 15 controls	14,4 (4,0) 15,3 (3,7)	fMRI: mental rotation task	Greater activity in the middle temporal, parietal, and lateral occipital cortices than in anterior cortical regions	Visuospatial deficit
Clements-Stephens et al. (45)	13 NF1 13 controls	9,80 (1,83) 9,78 (2,56); r: 7-15	fMRI: blocked paradigm (visual discrimination task)	Left hemisphere volume of activation greater than right across the frontal lobe and in posterior regions	Lower scores on Benton's JLO
Shilyansky et al. (46)	14 NF1 12 controls	24 (4,93) 22,58 (4,56)	fMRI: visuospatial working memory task	Hypoactivation of DLPFC, FEF, parietal cortex	Impairment in working memory maintenance task
Pride et al. (47)	25 NF1 18 controls	10,5 (7,3) 10,6 (2,9); r: 7-16	fMRI: Go/No-Go	Hypoactivation of pre-SMA, IFG, IOG and the fusiform gyrus/posterior cerebellum	Lower inhibition
Pride et al. (48)	19 NF1 18 controls	11,0 (2,8) 10,5 (2,5); r: 7-16	fMRI: auditory oddball processing	Hypoactivation in the ACC	Selective attention and attentional control
Ibrahim et al. (49)	23 NF1 25 controls	32,69 (9,03) 33,08 (8,89); r: 18-47	fMRI: spatial capacity working memory task	Hypoactivation of right IPS and left DLPFC (working memory circuitry). Greater connectivity between bilateral parietal regions and visual cortices, especially in left hemisphere, and lower connectivity between left temporal regions and PCC	Lower score in working memory task
Loitfelder et al. (50)	14 NF1 30 controls	12,49 (2,65) 12,30 (2,94)	fMRI: resting state	Positive coupling between left vACC and the frontal pole and the left amygdala and the right OFC	Worse executive, social and behavioral performance (no IQ correlation)
Violante et al. (51)	15 NF1 children and 13 NF1 adults 24 control children and 15 control adults	11,7 (2,9); r: 7-17 and 33,1 (4,9); r: 25-42 12,0 (2,3); r: 7-16 and 32,7 (5,6); r: 26-44	fMRI: blocked paradigm (low level visual stimulation)	Hypoactivation of low-level visual cortex. Failure to deactivate DMN during low level visual stimulation	Visuospatial deficit
Ribeiro et al. (52)	16 NF1 16 controls	14,1 (2,7); r: 10,2-19,7 13,8 (2,7); r: 10,4-19,5	fMRI: Go/No-Go task + EEG data + MRS (Ratios GABA/Cr and Glutamate/Cr)	Lower ratio GABA/Cr in the medial frontal reduced in frontal cortices	Inhibitory control: greater number of errors commission and faster reaction times in go trials indicating an impulsive response style
Jonas et al. (53)	29 NF1 22 controls	11,93 (2,64); r: 8-16 12,73 (3,49); r: 8-19	fMRI: Cake Gambling Task	Decreased neural activity in multiple regions including PCC and frontal pole	Risky decision making: non significant tendency to make fewer risky decisions across all reward categories

*ACC, anterior cingulate cortex; Cr, creatinine; DLPFC, dorsolateral prefrontal cortex; DMN, default mode network; EEG, electroencephalogram; FEF, frontal eye field; fMRI, functional magnetic resonance imaging; GABA, gamma-aminobutyric acid; IFG, inferior frontal gyrus; IQ, intellectual quotient; IOG, inferior occipital gyrus; IPS, intraparietal sulcus; JLO, judgement line orientation; MRS, magnetic resonance spectroscopy; NF1, neurofibromatosis type 1; PCC, posterior cingulate cortex; pre-SMA, pre-supplementary motor area; r, range; SD, standard deviation; STG, superior temporal gyrus, OFC, orbitofrontal cortex, vACC, ventral anterior cingulate cortex*.

#### Post-2010 Studies

##### PET

To our knowledge, since 2010 only one PET study by Apostolova et al. (54) using PET FDG in a large population (compared with previous studies) of 50 adult NF1 patients and 50 controls showed a single 11.2 ml cluster of reduced FDG uptake in the thalamus of NF1 patients compared with the control.

##### fMRI

Several research lines related to fMRI studies have been developed.

*The relationship between cerebral dysfunction and cognitive deficit*. Some studies have tried to link cerebral dysfunction and deficient cognitive processes in NF1. For example, North's team tried to correlate executive deficit, one of the characteristic deficits in NF1, and brain dysfunction. Pride et al. (47) (Table 3), using a Go/No-Go task to explore response inhibition, showed reduced activation compared to controls in the pre-motor and pre-supplementary motor area, the right anterior cingulate cortex, the right inferior frontal gyrus, the inferior occipital gyrus and the left fusiform gyrus. The literature identifies a relationship between impulsivity and sustained attention deficit, and hypoactivation of the right inferior frontal gyrus, known to be involved in the inhibition response (55). In a second study, Pride et al. (48) (Table 3) used an region of interest (ROI) approach focused on the attentional networks to identify hypoactivation in the exogenous attention system or bottom-up or ventral attention system during an auditory attention task. This network included the bilateral temporoparietal junctions and the anterior cingulate cortex. Moreover, the authors showed a correlation between brain activation level in the right inferior frontal gyrus and attention scores during the task.

*Functional connectivity*. Due to the known abnormalities in the macro and micro structures of white matter, several groups have investigated the possible abnormalities in functional connectivity of the brain of NF1 subjects. In adults, Ibrahim et al. (49) (Table 3) showed reduced recruitment of the left dorsolateral prefrontal cortex (DLPFC) and parietal cortex during a visuospatial working memory task, which confirms previous results by Shilyansky et al. (46). The authors also found differences in the task-related functional connectivity between NF1 subjects and control subjects: during a visuospatial working memory task they observed greater connectivity between bilateral parietal regions and the visual cortex and lower connectivity between the posterior cingulate cortex and the left temporal region in NF1 subjects compared to controls. These connectivity differences suggest an inactivation deficit of the default mode network (DMN) in NF1 subjects, an inactivation that usually occurs during cognitive tasks. This confirms the results of a previous study in children conducted by Loitfelder et al. (50) (Table 3). In this study, functional imaging data were collected during resting state fMRI (rs-fMRI) and cognitive and social skills were evaluated with parent questionnaires (BRIEF, DEX, SRS, Social Skills Rating system: SSRS, CBCL). Among the more significant results, the authors showed an increase in connectivity between the left ventral anterior cingulate cortex and the frontal cortex, between the left amygdala and the posterior cingulate cortex/precuneus, and between the left orbito-frontal cortex and the homolateral pallidum in NF1 children compared to controls. Using rs-fMRI, Tomson et al. (56) found a reduction in postero-anterior “long distance” connectivity in NF1 subjects compared to controls along with a less organized DMN and visual network.

ADHD is frequent in the NF1 population and Jonas et al. (53) consequently explored the hypothesis of a deficit in reward processing since this has been shown in ADHD patients without NF1 (Table 3). Neuroimaging revealed reduced neuronal activity in the regions involved in the reward circuitry (anterior cingulate, paracingulate, supramarginal, and angular gyri) and a different blood oxygenation level dependent (BOLD) signal development across ages between NF1 subjects and controls, especially in frontal regions, with a decrease in neural activity related to an increase in age in the controls and an increase in neural activity related to an increase in age in the patients with NF1.

*Multimodal approach*. Violante et al. (51) focused on the analysis of visuoperceptual deficit in NF1 (Table 3). To this end, the authors used fMRI with a block design, including a rest period and a different visual stimulus presentation that stimulated either magnocellular or parvocellular pathways: M stimuli (25 cycles per degree, 18 Hz, low contrast: 18%) and P stimuli (2 cycles per degree, 2 Hz, high contrast: 100%). They used an ROI approach that focused on the occipital lobe: areas V1, V2, and V3. The main results were: a functional deficit for low-level stimuli, magno- or parvocellular, in children as well as adults; greater hypoactivation in the extrastriate cortex (V2 and V3) of the dorsal pathway, and abnormal activation during low-level M stimuli (suggesting an interference by deficient deactivation) in the DMN (medial prefrontal cortex, anterior cingulate cortex (ACC), the posterior parietal cortex and parietal cortex) related to ADHD frequency in this population. To pursue the physiopathological analysis of the visual cortex in NF1 patients, the same team conducted a study that combined fMRI, spectroscopy (GABA/Creatinine and Glutamate/Creatinine ratios in the occipital cortex) and genetic analysis (57). Eighteen NF1 children and 26 controls aged 7 to 19 years performed a simple fMRI visual task in which they had to press a button whenever a target disappeared. Through the results, the authors were able to show a reduction in the GABA/Creatinine ratio in NF1 subjects compared to the controls but no difference in the Glutamate/Creatinine ratio, which suggests an alteration in the balance between excitatory and inhibitory mechanisms with altered inhibition in the NF1 occipital cortex. The authors also found a correlation between mutation type and GABA level suggesting a role of neurofibromin in GABAergic neurotransmission. Lastly, they showed a negative correlation between GABA/Creatinine and BOLD level with no difference between the NF1 subjects and the controls. The same team [Table 3; Ribeiro et al. (52)], using combined high-density electroencephalography, MRS and fMRI with a Go/No Go task, examined the neural mechanisms of impulsive behavior in NF1. During the Go/No Go task in visual modality, NF1 subjects made more errors of omission and had a faster reaction time, which confirms the impulsive phenotype. This behavior was correlated with a decrease in GABA/Creatinine ratio found in the medial frontal cortex (including the pre-motor area, the supplementary motor area and the ACC). However, the decrease in this same ratio in the occipital regions was not correlated with the behavioral data. Regarding evoked potentials, an early component corresponding to early visual processing and a later component in the frontal regions matched the inhibitory response that was altered in NF1 subjects. However, in this study, there was no link between genetic findings and altered GABAergic neurotransmission in frontal regions.

*Evaluation of the therapeutic care of NF1 patients*. In conclusion with regards to functional neuroimaging, two research teams used fMRI as a means to evaluate NF1 patient therapy. The first research, conducted by Charbernaud et al. (58), did not include a control group, which limits the interpretation of the results. In this phase 1, open label trial including 7 children aged 10 to 15 years treated with lovastatin for 12 weeks, an MRI was performed 1 day prior to the start of treatment and on the last day of treatment. The authors compared functional activity in rs-fMRI on the first neuroimaging and functional activity in the resting block between periods of visual stimuli on the second MRI, which they considered as a “pseudo rs-fMRI.” The main result was an increase in anterior-posterior long-range connectivity and a decrease in short-range connectivity as observed in normal development. A second preliminary study, conducted by Yoncheva et al. (59) in 16 NF1 children aged 8 to 15 years with a deficit in working memory, evaluated the impact of cognitive training (Cogmed Training) in 25 sessions for 6 and 10 weeks. An rs-fMRI was performed before and after training. Four rs-fMRI indices previously used in typically developing children were analyzed: the amplitude of low frequency fluctuations (ALFF) and the fractional amplitude of low frequency fluctuations (fALFF) that characterize intrinsic neural activity, regional homogeneity (ReHo) that characterizes local synchronization and voxel mirrored homotopic connectivity (VMHC) that reflects interhemispheric synchronization. After training a reduction in fALFF in the cerebellum (left cerebellum I to IV and right cerebellum V) and in the thalamus (right and left), the authors observed a reduction in ReHo in the right middle frontal gyrus and an increase in ReHO in the left fusiform gyrus. This was a preliminary study that showed the possibility to record neural activity changes after training, but this did not enable us to distinguish a developmental effect vs. a specific effect of training because of the absence of a control group.

#### Summary

Functional neuroimaging studies have primarily explored two main characteristics of the NF1 cognitive phenotype: executive function deficit and visuospatial deficit.

The deficit in executive functions is characterized by deficient inhibitory control and a deficit in exogenous attention depending on the external stimuli. It appears to be associated with a dysfunction in the right inferior frontal areas and the middle frontal areas (pre-motor area, supplementary motor area and ACC). A GABAergic deficit has also been shown for these regions.

Concerning the visuospatial deficit, studies have suggested a dysfunction in the visual cortex (V2-V3) and especially in the magnocellular pathway involved in the processing of low spatial frequency and high temporal frequency. This dysfunction could be associated with a disruption in excitatory-inhibitory balance, which involves neurofibromin, with a decrease in inhibitory GABA in the occipital cortex in NF1.

Studies that specifically address the issue of connectivity show an altered neural connectivity in NF1 subjects compared to controls with a reduction in anterior-posterior “long-range” connectivity and a deficit in deactivation in DMN during cognitive tasks.

Abnormalities observed in brain activity can serve as a basis to evaluate the efficacy of new therapeutics that could be used in NF1 subjects over the next few years. Preliminary studies suggest the possibility to use this technique as a biomarker in future treatment trials.

Characteristics of the main neuroimaging studies showing a link between functional features and cognitive functions are indicated in the Table 3.

## Conclusion

**Figure 1:**
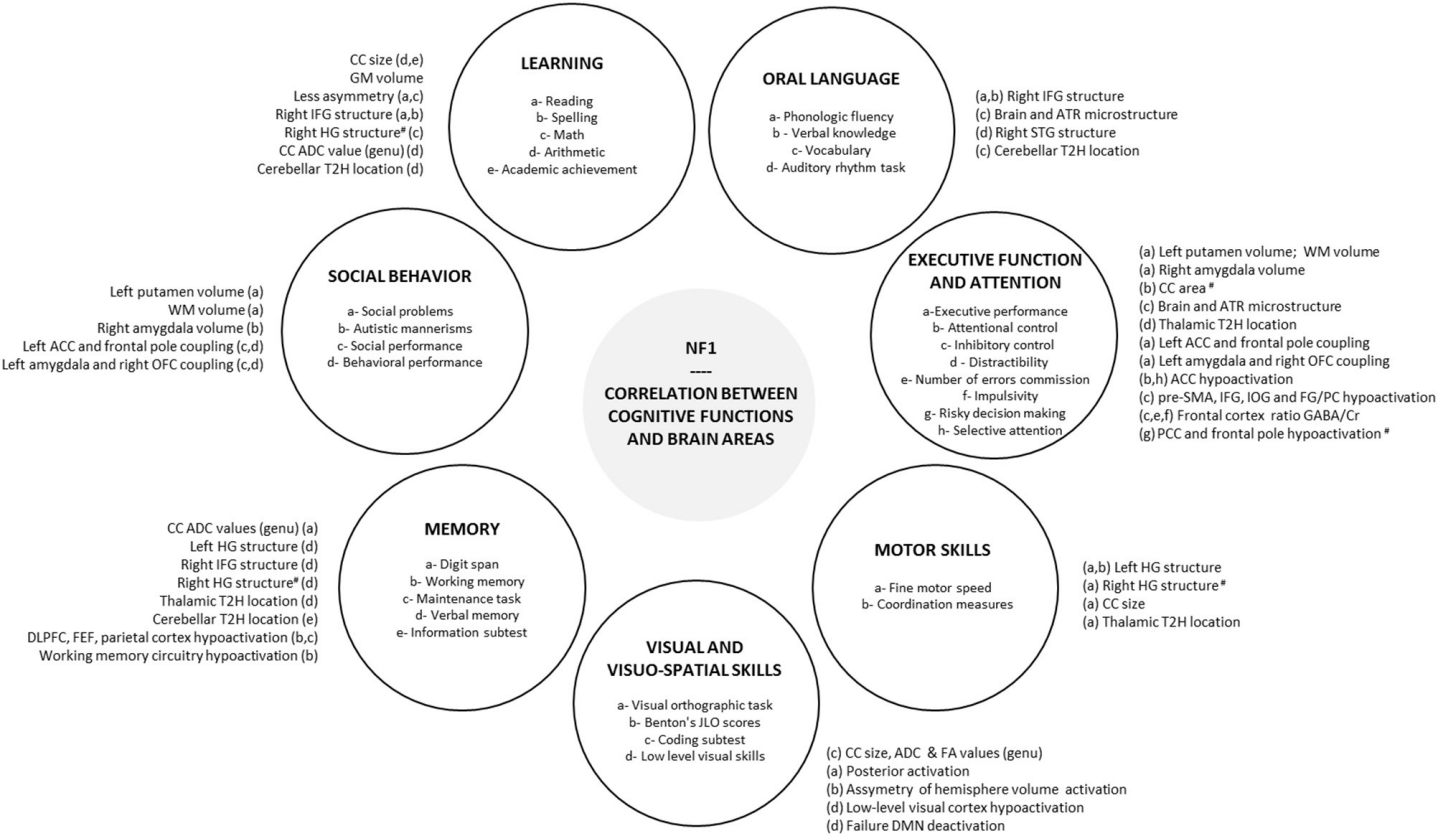
Correlation between cognitive functions and brain areas. Correlations reported in the literature between brain areas and each cognitive and behavioral functions impacted in NF1 are summarized in peripheral circles. #, negative correlation; ACC, anterior cingulate cortex; ADC, apparent diffusion coefficient; ATR, anterior thalamic radiation; CC, corpus callosum; Cr, creatinine; DLPFC, dorsolateral prefrontal cortex; DMN, default mode network; EF, executive function; FA, fractional anisotropy; FEF, frontal eye field; FG, fusiform gyrus; GABA, gamma-aminobutyric acid; HG, Heschl's gyrus; IFG, inferior frontal gyrus; IOG, inferior occipital gyrus; JLO, judgement line orientation; NF1, neurofibromatosis type 1; OFC, orbitofrontal cortex; PC, posterior cerebellum; PCC, posterior cingulate cortex; pre-SMA, pre-supplementary motor area; SLF, superior longitudinal fasciculus; STG, superior temporal gyrus; T2H, T2 hyperintensity, WM, white matter.

Although the link between the specific cognitive and behavioral features of NF1 and cerebral characteristics is not totally clear at present, comprehension of the neural basis has improved thanks to emerging neuroimaging methods. Executive dysfunction in NF1 children seems to be associated with a dysfunction in the right inferior frontal areas and the middle frontal areas and an alteration in microstructural integrity (DTI) in ATR. Visuospatial deficit appears to be correlated with a dysfunction in the visual cortex (V2-V3) and especially in the magnocellular pathway involved in the processing of low spatial frequency and high temporal frequency. Moreover, connectivity studies show an altered neural connectivity with a reduction in anterior-posterior “long-range” connectivity and a deficit in deactivation in DMN during cognitive tasks. Therefore, functional MRI has become a widely used technique over the past years and might be helpful in the understanding of the cerebral basis of NF1 cognitive phenotype. However, at present there are inconsistencies in the findings of several studies with regard to morphological and macrostructural neuroimaging brain features (UBOs, megalencephaly, higher volume of sub-cortical structures) in NF1. Therefore, it is difficult to find a conclusive link between these features and neurocognitive phenotype (Figure 1).

Recently, a study by our group using a multimodal approach involving measures of gray matter volume, fractional anisotropy, and mean diffusivity highlighted a NF1 brain signature (11). Considering that studies using a monomodal approach have failed to explain the cognitive phenotype in NF1, in the future, the development of multimodal approaches could help to clarify the relationships with NF1 phenotype and evaluate the efficacy of specific therapeutics. Moreover, the cognitive phenotype of NF1 subjects is extremely variable from one individual to another. This heterogeneity is probably multifactorial resulting from genetic and environmental factors in such a way that an approach exclusively based on neuroimaging cannot entirely explain the cognitive phenotype. The study of the impact of various factors that influence the cognitive phenotype (genetic, environmental, etc.) remains an indispensable complement to the neuroimaging approach in NF1.

## Author Contributions

YC wrote the first draft of the manuscript. EB translated and completed a part of the manuscript, and drew up the tables. FN made critical amendments and gave essential feedback for the manuscript. MB developed and formatted the figure and helped to improve tables. SM gave essential feedback for the manuscript. PP made critical amendments especially for the figure. All authors read and approved the final manuscript.

### Conflict of Interest

The authors declare that the research was conducted in the absence of any commercial or financial relationships that could be construed as a potential conflict of interest.

## Abbreviations

ACC: anterior cingulate cortex
AD: axial diffusion
ADC: apparent diffusion coefficient
ADHD: Attention Deficit and Hyperactivity Disorder
ASD: Autism Spectrum Disorder
ATR: anterior thalamic radiation
BRIEF: Behavioral Rating Inventory of Executive Function
CBCL: Child Behavior Check List
CC: corpus callosum
DLPFC: dorsolateral prefrontal cortex
DEX: Dysexecutive Questionnaire
DMN: default mode network
DTI: diffusion tensor imaging
FA: fractional anisotropy
FDG: fluorodeoxyglucose
FEF: frontal eye fields
FLAIR: Fluid-Attenuated Inversion Recovery
fMRI: functional magnetic resonance imaging
GABA: gamma-aminobutyric acid
GM: gray matter
IQ: intelligence quotient
JLO: Judgment of Line Orientation
MD: mean diffusivity
MRI: magnetic resonance imaging
MRS: magnetic resonance spectroscopy
NAA: N-acetyl-aspartate
NF1: Neurofibromatosis type 1
PET: positron emission tomography
RD: radial diffusion
ROI: region of interest
SRS: Social Responsiveness Scale
UBOs: unidentified bright objects
WM: white matter.
